# The dynamics of dementia communication

**DOI:** 10.1177/1471301220985404

**Published:** 2021-02-08

**Authors:** Catherine T Parsons

**Affiliations:** 3660 University of Surrey

Living with dementia has many challenges. Each person is unique and the experience of dementia will differ from person to person, but one challenging aspect of dementia, potentially one of the most distressing aspects, is the impact it has on communication. Many people living with dementia experience difficulties in communicating their thoughts and needs and interpreting the communication of others. This can be distressing both for the person with dementia and for their family, friends and carers.

In *The Dynamics of Dementia Communication*, Alison Wray takes on the immense task of exploring why communication is so negatively impacted by dementia; why it makes living with dementia so difficult; and what approaches to communication work best and why.

The book is aimed at anyone who is interested in the difficulties associated with communication and dementia – academics, practitioners, friends, family, carers and people living with dementia. As more than 850,000 people are living with dementia in the UK, it is likely that this book will have relevance to many people as dementia touches the lives of so many professionally and/or personally. I am a researcher in dementia care, I have worked as a social work practitioner, and my father has dementia. These multiple perspectives informed my review of this book.

The book is well laid out, with the first section looking at the contexts which shape communication, the second focussing on conceptualizing communication and the third looking at applications and implications. The chapters can be read individually, which is useful for those with limited time or interest in only certain aspects. There is good use of diagrams, making it easier to understand sometimes difficult concepts and many real-life examples, which brings the subject to life.

Professor Wray covers the biological, social and emotional factors associated with the experience of living with dementia and discusses the different care approaches including person-centred care and relationship-centred care, acknowledging that not all care approaches have been covered. It was good to see inclusion and discussion of many of the seminal names associated with dementia care such as Kitwood and Sabat alongside discussion of more recent ideas such as dementia villages and also input from people living with dementia such as Wendy Mitchell. In this respect, her book whilst interesting and comprehensive is similar to many other books on dementia, but it is the emphasis on communication and the introduction of the Communicative Impact model where Professor Wray with her experience and expertise in language and communication really adds to the dementia debate.

Professor Wray proposes that the basic function of communication, including language, is to help us change our world in a way which is beneficial to our survival, and this includes using it to improve our quality of life and helping us sustain valued relationships. Professor Wray argues that dementia destabilises the basic dynamics which make communication work, in particular around shared contextual knowledge and the assumptions we make about what the other person means. This can affect communication impact. When we have anxieties related to whether we have achieved communication impact, either as a person with dementia or as an interlocutor, this can result in defensive responses such as fear or anger. A person with dementia may have to change their behaviour or their interlocutor may have to, in order to create a new communicative space for mutual use.

The Communicative Impact model is not specific to dementia, but Professor Wray argues it can be used to interpret the relationship between the manifestations of dementia and the experience of those living with dementia and can be useful in identifying, exploring and explaining communication problems.

My favourite section of the book is the last section which looks at the applications and implications. In this section, Professor Wray explores how people living with dementia are perceived, whether it is acceptable to deceive them and the communication approaches most likely to be effective, using the Communicative Impact model as a lens through which to interpret them.

The exploration on whether a person with dementia is viewed as different by “degree” or “kind” was particularly interesting. The exploration of the reasons why a carer might adopt a certain stance and the emotional impact on them made interesting reading.

The discussion on truth telling in a dementia care context was enlightening, and certainly I had not considered some of the examples given as potential examples of deception. Is a 1950s style fake bus stop in a care home an example of emotional manipulation? This discussion provided much to think about and consider. Professor Wray suggests we need to look at the reasons people lie – is it out of kindness or for social control – and explore whose Communicative Impact is prioritised.

The book ends with an agenda for improving communication in the dementia context. Professor Wray argues that communication is a principle component of compassionate person-centred care and the communication acts of people with dementia need to be viewed as instances of real engagement. Some environments are more conducive for rewarding communication than others and we must be aware of that, but we must always ask ourselves, what is the person with dementia aiming to achieve. There are recommendations for practice to enable carers to find alternative ways to assist communication. There are also policy level recommendations where Professor Wray argues that social reserve must be promoted through more and better trained staff as well as through improved joined-up health and social care infrastructure.

The only area I would have liked to see more exploration of in this book is the use of technology to aid communication. Professor Wray does mention this, and there is note of Virtual Reality Apps to encourage empathy by showing people what it is like to live with dementia; I feel this growing area of interest could have been discussed further.

Professor Wray notes that the essence of the practices commended in the book is to act with (true) kindness. The emphasis on kindness and compassion throughout the book is obvious and good to see.

As a researcher, a practitioner and a family member of a person with dementia, I found this book helpful and inspiring and it led to me thinking differently in how I view communication difficulties which can be so challenging and distressing for those who are living with dementia and those who love and care for them. I recommend this book to all whose lives are touched by dementia.

